# Дисфункциональные липопротеины высокой плотности при сахарном диабете 2 типа

**DOI:** 10.14341/probl13118

**Published:** 2022-06-10

**Authors:** О. Н. Потеряева, И. Ф. Усынин

**Affiliations:** Научно-исследовательский институт биохимии Федерального исследовательского центра фундаментальной и трансляционной медицины; Научно-исследовательский институт биохимии Федерального исследовательского центра фундаментальной и трансляционной медицины

**Keywords:** липопротеины высокой плотности, сахарный диабет 2 типа, модификация структуры, дисфункция, обзор

## Abstract

Риск развития сердечно-сосудистых заболеваний (ССЗ) у лиц с сахарным диабетом 2 типа (СД2) увеличивается в 2–4 раза. Одним из основных факторов повышения сердечно-сосудистого риска является дислипидемия, которая включает аномалии во всех липопротеинах, в том числе липопротеинах высокой плотности (ЛПВП). Развитие СД2 сопровождается не только снижением уровня ЛПВП, но и существенными изменениями в их структуре. Это приводит к трансформации нативных ЛПВП в так называемые дисфункциональные, или диабетические, ЛПВП, которые утрачивают свои антиатерогенные, кардиопротективные, противовоспалительные и антидиабетические свойства. При плохо контролируемом диабете ЛПВП могут не только терять свои полезные функции, но и приобретать проатерогенные, провоспалительные. Диабетические ЛПВП могут способствовать развитию таких неблагоприятных процессов, как усиление пролиферации, миграции и инвазии клеток рака. Учитывая, что ЛПВП, помимо участия в транспорте холестерина, выполняют в организме важные регуляторные функции, есть основание предполагать, что структурные модификации ЛПВП (окисление, гликирование, обогащение триглицеридами, потеря ЛПВП-ассоциированных ферментов и др.) являются одной из причин развития сосудистых осложнений диабета.

## Abstract (en)

The risk of cardiovascular disease (CVD) in persons with type 2 diabetes mellitus (DM2) increases two to four times. One of the main factors increasing cardiovascular risk is dyslipidemia, which includes abnormalities in all lipoproteins, including high-density lipoproteins (HDL). The development of DM2 is accompanied not only by a decrease in the level of HDL, but also by significant changes in their structure. This leads to the transformation of native HDL into so-called dysfunctional or diabetic HDL, which loses their antiatherogenic, cardioprotective, anti-inflammatory and anti-diabetic properties. In poorly controlled diabetes mellitus HDL can not only lose its beneficial functions, but also acquire proatherogenic, proinflammatory ones. Diabetic HDL can contribute to the accumulation of such unfavorable qualities as increased proliferation, migration, and invasion of cancer cells. Given that HDL, in addition to participation in cholesterol transport, performs important regulatory functions in the body, there is reason to assume that structural modifications of HDL (oxidation, glycation, triglyceride enrichment, loss of HDL-associated enzymes, etc.) are one of the causes of vascular complications of diabetes.

## ВВЕДЕНИЕ

Общая численность пациентов с сахарным диабетом (СД) в РФ на январь 2021 г. составила 4 799 552 (3,23% населения РФ), из них доля СД 2 типа  — 92,5% (4,43 млн). Основной причиной высокой смертности при СД являются сердечно-сосудистые заболевания (ССЗ) [1]. Развитие ССЗ у лиц с СД 2 типа (СД2) увеличивается в 2–4 раза [2]. Одним из основных факторов повышения сердечно-сосудистого риска, связанного с диабетом, является дислипидемия, которая включает в себя аномалии во всех фракциях липопротеинов, в том числе липопротеинов высокой плотности (ЛПВП) [3]. Дислипидемия развивается у 72–85% больных, изменения липидного спектра часто опережают развитие СД2 на несколько лет [4].

Крупные эпидемиологические исследования продемонстрировали обратную связь между концентрацией сывороточного холестерина (ХС) ЛПВП (ХС-ЛПВП) и риском развития ишемической болезни сердца (ИБС). Каждое увеличение ХС-ЛПВП на 0,026 ммоль/л снижает риск развития ИБС на 2–3% [5][6]. Пациенты с низким уровнем ЛПВП в 2 раза чаще страдают СД и имеют более высокий риск развития связанных с ним сердечно-сосудистых осложнений, периферической невропатии и диабетической нефропатии [7][8].

ЛПВП противодействуют нарушениям метаболизма на фоне СД2. Они обладают потенциальными антидиабетическими свойствами, что подтверждается экспериментальными исследованиями: ЛПВП увеличивают поглощение глюкозы скелетными мышцами и стимулируют синтез и секрецию инсулина изолированными островками Лангерганса поджелудочной железы [9][10]; ингибируют апоптоз β-клеток [8][10]; повышают чувствительность периферических тканей к инсулину [11]. Введение человеческого апоА-I инсулинорезистентным мышам приводило к значительному улучшению секреции инсулина и стимуляции поглощения глюкозы скелетными мышцами [12][13]. Этот терапевтический потенциал был подтвержден в исследовании у пациентов с СД2 [11].

Помимо прямого воздействия на метаболизм глюкозы, ЛПВП влияют на обратный транспорт ХС из артериальной стенки и периферических тканей в печень; предохраняют липопротеины низкой плотности (ЛПНП) от окисления; оказывают противовоспалительное и сосудорасширяющее действия на клетки сосудистой стенки [6][8].

В настоящее время растет количество фактов, свидетельствующих о том, что у модифицированных ЛПВП нарушается способность к обратному транспорту ХС и они утрачивают свои атеропротективные свойства [8][14]. Более того, при плохо контролируемом СД2 ЛПВП могут терять свои полезные функции и приобретать проатерогенные, провоспалительные свойства. Такие ЛПВП принято называть дисфункциональными, а в случае СД  — диабетическиеми ЛПВП [6][8][15][16].

## 1. Нарушение структуры ЛПВП при СД2

У больных СД2 происходят количественные изменения в спектре липопротеинов: снижается уровень ЛПВП, ХС-ЛПВП и аполипопротеина А-I (апоА-I), повышается концентрация апоВ (основного белка ЛПНП и липопротеинов очень низкой плотности (ЛПОНП)). Наибольшие уровни апоВ, индекса атерогенности были отмечены в группе больных с высоким содержанием триацилглицеридов (ТАГ) в сыворотке крови. У 80% больных СД2 ЛПВП обогащаются ТАГ, содержание которых в ЛПВП может достигать 2,6 ммоль/л [3][17]. Самые низкие уровни ХС-ЛПВП отмечались у лиц с плохо контролируемым СД2 и высоким уровнем гликированного гемоглобина (HbA1c) [18]. Низкие уровни ХС-ЛПВП являются наиболее частыми нарушениями, наблюдаемыми у мужчин с СД2 [19].

Частицы ЛПВП при СД2 претерпевают качественные изменения, включающие обогащение ТАГ, истощение эфиров ХС, конформационные изменения апоА-I, гликирование или окислительную модификацию аполипопротеинов, липидов и/или ЛПВП-ассоциированных ферментов. Замена эфиров ХС на ТАГ в липидном ядре ЛПВП ведет к снижению проникновения центральных и С-концевых областей апоА-1 в липидную фазу, увеличивая доступность аминокислотных остатков, в частности метионина, для липидных перекисей. Потеря эфиров ХС приводит к утрате конформационной устойчивости апоA-I и образованию нестабильных частиц, которые быстрее выводятся из кровообращения. Предполагают, что соотношение эфиров ХС/ТАГ вЛПВП является ключевым фактором, определяющим время их пребывания в крови [20].

Так как диабет связан с длительным хроническим воспалением, у пациентов с СД2 происходит замена aпoA-I на провоспалительный белок острой фазы SAA (Serum Amyloid A), который транспортируется в небольших фракциях ЛПВП и легко вытесняет апоА-I и другие аполипопротеины с поверхности частиц (до 86% от общего белка ЛПВП). Замена апоА-I на SAA способствует ускоренному выведению ЛПВП из кровообращения, повышает связывание ЛПВП с протеогликанами артериальной стенки [21].

У пациентов с СД2 снижается содержание апоЕ в ЛПВП, что ухудшает отток ХС из макрофагов человека к ЛПВП и усиливает связывание ЛПНП со стенкой сосуда. Уменьшение частиц ЛПВП, содержащих апоM и богатых сфингозин-1-фосфатом (S1P), препятствует расширению артериальных сосудов за счет снижения продукции эндотелиального оксида азота [16].

Изменение фосфолипидного состава ЛПВП описано при развитии преддиабета и СД2. Увеличение фосфолипидов, основных носителей сильно окисляемых полиненасыщенных жирных кислот, в частицах ЛПВП повышает в них окислительные процессы и снижает их способность предотвращать окисление ЛПНП [22]. Повышение уровня церамида, провоспалительного липида, участвующего в развитии инсулинорезистентности скелетных мышц и воспалении, губительно для β-клеток поджелудочной железы [23].

Гликирование ЛПВП

Хроническая гипергликемия, наблюдаемая при СД2, приводит к процессу гликозилирования белков, т.е. ферментативному взаимодействию белков с углеводами. Процесс протекает в эндоплазматической сети клетки с участием ферментов (гликозилтрансфераз), катализирующих наращивание олигосахаридной цепи на молекуле белка, и заканчивается образованием полноценного гликопротеина. Однако в организме человека может происходить процесс неферментативного гликозилирования (гликирования) как результат реакции Майяра [24]. Гликирование может протекать в тканях здоровых людей, но с большей скоростью происходит у лиц с гипергликемией. Неферментативному гликированию in vivo подвержены многие белки, включая апопротеины всех классов липопротеинов, в том числе основные белки ЛПВП [21]. Вначале между глюкозой и свободными аминогруппами аполипопротеинов образуется нестабильная альдиминовая группировка, которая впоследствии превращается в более стабильные соединения, так называемые ранние продукты гликирования (основания Шиффа, продукты Амадори). Их дальнейшее превращение приводит к образованию необратимых конечных продуктов гликирования (КПГ). Более быстрое образование КПГ происходит из дикарбонильных предшественников, синтезированных внутриклеточно из глюкозы: глиоксаль, метилглиоксаль, 3-дезокиглюкозон. Глиоксаль образуется в результате аутоокисления глюкозы; метилглиоксаль возникает при фрагментации глицеральдегид-3-фосфата в процессе гликолиза; 3-дезокиглюкозон  — при распаде продукта Амадори [24].

Метилглиоксаль (альдегид пировиноградной кислоты), реакционноспособное карбонильное соединение, считается одним из важнейших реагентов гликирования, ковалентно связывающихся с аминогруппами белков. Концентрация метилглиоксаля в плазме крови у лиц с СД достигает 0,4–0,5 ммоль/л независимо от уровня гликемического контроля. В процессе гликирования in vitro наблюдалось снижение свободных аминогрупп апоА-I, изменялся поверхностный заряд белка, что приводило к значительному снижению его аффинности к фосфолипидным везикулам [25]. Кроме того, в среде инкубации увеличивалось количество флуоресцентных КПГ, значительно снижалась активность фермента антиоксидантной защиты  — параоксаназы-1 (ПОН-1), который преимущественно связан с ЛПВП. Показано, что триптофан и цистеин, ключевые компоненты активного центра ПОН-1, являются мишенями для метилглиоксаля. Их гликирование чаще всего становится причиной низкой активности ПОН-1 [25][26].

У больных с плохо контролируемым СД2 гликирование способствовало ковалентному связыванию оксоальдегидов с остатками лизина, аргинина и цистеина апоА-1, а также с N-концевыми аминогруппами апобелка, что приводило к формированию устойчивых меж- и внутримолекулярных поперечных сшивок и изменяло конформацию апоА-1. Последнее снижало доступность моноклональных антител к апоА-I [27]. В процессе гликирования апоA-I наблюдали образование димеров, тримеров или гетеродимеров с другими аполипопротеинами (например, апоA-I-апоA-II) или других агрегатов с более высокой молекулярной массой [26]. У пациентов с СД наблюдалось увеличение на 50–70% аддуктов ЛПВП, полученных из метилглиоксаля. Метилглиоксаль и гликолевый альдегид модифицировали боковые цепи лизина и аргинина человеческого рекомбинантного апоА-I, изменяли вторичную структуру апобелка, увеличивая содержание α-спиралей. Гликированный апoA-I, выделенный отпациентов с СД2, утрачивал липидсвязывающую способность за счет уменьшения количества положительно заряженных боковых цепей лизина и аргинина, что снижало его взаимодействие с отрицательно заряженными фосфолипидными головками [27].

Гликирование ЛПВП у больных с СД2 приводило к диссоциации апоА-I от частицы ЛПВП, после чего делипидированный апобелок подвергался поглощению и деградации в проксимальных почечных канальцах. Период полураспада гликированного апоA-I в 3 раза короче, чем нативного белка. Между гликированным гемоглобином и скоростью катаболизма апоА-I была обнаружена сильная корреляционная связь [28].

Окисление в ЛПВП

Повышенный уровень глюкозы в эндотелиальных клетках запускает внутриклеточное образование КПГ, которые индуцируют выработку активных форм кислорода, что инициирует окисление ЛПВП. Многие исследователи рассматривают окислительный стресс, индуцированный гипергликемией, как основной механизм повреждения β-клеток и прогрессирования СД [2]. Клетки Caco-2, обработанные средой с высоким содержанием глюкозы (50 мМ), увеличивали продукцию активных форм кислорода, усиливали процессы перекисного окисления липидов и образования КПГ [29].

ЛПВП in vitro легко модифицируются различными окислителями, такими как ионы металлов, пероксильные и гидроксильные радикалы, альдегиды, липооксигеназы, сигаретный дым. Под действием окислителей изменяется как поверхностный слой частицы ЛПВП (белки, фосфолипиды и ХС), так и гидрофобное ядро (эфиры ХС). В аполипопротеинах ЛПВП окислению подвергаются аминокислотные остатки метионина, цистеина, тирозина и лизина [21]. Аминокислотные остатки метионина, расположенные в положениях 112 и 148 структуры апоA-I, определяют антиоксидантную способность ЛПВП, приводящую к снижению пероксидных радикалов (LOO•) или пероксид-липидов (LOOH) до редокс-неактивных гидроксидов (LOH), завершая цепные реакции перекисного окисления липидов. У пациентов с СД содержание окисленных остатков метионина в апоА-I повышено в положениях 86, 112 и 148, в результате происходит снижение активности ПОН-1 в ЛПВП [20].

В частицах ЛПВП пациентов с СД2 увеличивались активность и количество фермента миелопероксидазы по сравнению со здоровыми лицами. Миелопероксидаза запускает процессы нитрования и хлорирования аминокислотных остатков апобелкови приводит к увеличению циркулирующего уровня окисленного апоА-I, наблюдаемого у пациентов с СД2 [21].

Активация белков, переносящих липиды

Гипергликемия и гликирование повышают уровень белка, переносящего эфиры ХС (БПЭХ). Активность БПЭХ, который в основном транспортируется в составе ЛПВП, повышена при СД2 [3]. При метаболическом синдроме и СД2 повышение активности БПЭХ приводит к увеличению переноса эфиров ХС от ЛПВП к богатым триглицеридами липопротеинам и реципрокного переноса ТАГ в ЛПВП, что сопровождается потерей апоА-I, в первую очередь из маленьких, плотных частиц [30]. Кроме того, недавнее исследование пациентов с СД показало, что гликирование апоC-I снижает его ингибирующее действие на БПЭХ. Сообщалось также об увеличении количества и активности белка-переносчика фосфолипидов, PLTP (Phospholipid transfer protein) у пациентов с СД2, что коррелировало с увеличением толщины интима-медиа сосудов [3]. Таким образом, повышение активности БПЭХ является проатерогенным у пациентов с СД2 [31].

Изменение гетерогенности частиц

Гетерогенность ЛПВП и профиль частиц в значительной степени отражают нарушения в метаболизме ЛПВП. Концентрация в плазме мелких, плотных частиц ЛПВП увеличивается при гиперхолестеринемии, гипертриглицеридемии, дефиците лецитин-холестерин ацилтрансферазы (ЛХАТ), а также у пациентов с ИБС и СД2 [3]. При дислипидемии метаболического синдрома и СД2 циркулирующие уровни больших сферических частиц ЛПВП, богатых ХС, снижаются параллельно со снижением ХС-ЛПВП, соответственно уменьшается содержание aпoA-I в крупных частицах ЛПВП2b и ЛПВП2a. В крови таких пациентов преобладают небольшие, плотные частицы ЛПВП3, что указывает на нарушение преобразования малых частиц в большие, сферические [16][21]. Кроме того, печеночная липаза, экспрессия и активность которой увеличиваются при гипергликемии и инсулинорезистентности, метаболизирует богатые триглицеридами ЛПВП, приводя к образованию мелких частиц ЛПВП и их ускоренному клиренсу [16]. При плохом гликемическом контроле у пациентов с СД2 частицы ЛПВП3c были единственной субфракцией, обогащенной ТАГ и фосфолипидами, но обедненной эфирами ХС и апопротеинами, при этом снижалась их функциональность. Корреляционный анализ показал, что эти изменения были связаны только с уровнями HbA1c [30]. Показано, что крупные частицы обладают более высокими антидиабетическими функциями по сравнению с малыми ЛПВП [10].

Таким образом, гипергликемия, активация окислительного стресса и хроническое воспаление, характерные для СД2, вызывают окислительную модификацию и гликирование белковых компонентов ЛПВП, повышают уровень БПЭХ, нарушают преобразования малых частиц в большие, сферические (рис. 1). Патологическая модификация ЛПВП приводит к их дисфункции: утрате защитных функций и приобретению проатерогенных и провоспалительных свойств даже при физиологическом уровне ЛПВП [5][32].

**Figure:**
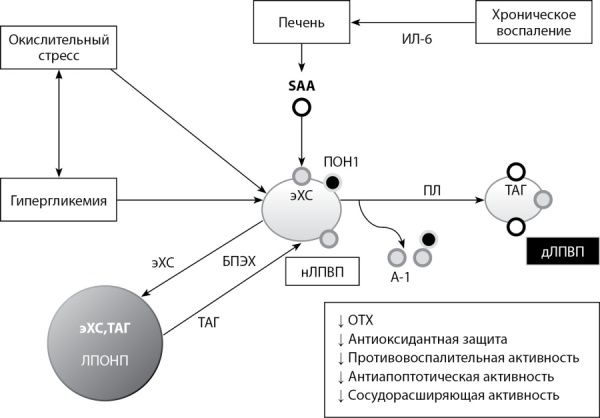
Рисунок 1. Нарушение структуры и функциональных свойств ЛПВП при СД2 (адаптировано из [21]).Figure 1. Structural and functional impairment of HDL in T2DM (adapted from [21]). Сокращения: нЛПВП  — нативные ЛПВП; дЛПВП  — дисфункциональные ЛПВП; эХС  — эфиры ХС; ПЛ  — печеночная липаза; ИЛ-6 — интерлейкин-6. Обогащение ЛПВП триглицеридами сопровождается конформационными изменениями в структуре aпoA-I; гипергликемия приводит к гликированию апоА-I; окислительный стресс модифицируют специфические аминокислотные остатки в апоА-I; хроническое воспаление сопровождается увеличением уровня ИЛ-6 в плазме крови и приводит к повышению продукции SAA, который замещает апоА-I и ферменты, связанные с ЛПВП; гидролиз с помощью печеночной липазы приводит к образованию небольших, плотных частиц ЛПВП, которые обогащены ТАГ и SAA; нативные частицы ЛПВП, подвергшиеся таким модификациям, теряют свои исходные функциональные свойства.

## 2. Нарушение функций диабетических ЛПВП

Первоначально связь между ЛПВП и некоторыми патологиями была исследована только на уровне количества ЛПВП и ХС-ЛПВП. Однако в последние годы акцент стали делать на состоянии функции ЛПВП, поскольку было показано, что некоторые патологии, такие как метаболический синдром, СД, ИБС, хронические заболевания почек, инфекции, вызывают функциональные изменения в ЛПВП [2][25].

Обратный транспорт холестерина

Обратный транспорт ХС является одной из ведущих функций ЛПВП [2][33]. Показано, что ЛПВП от пациентов с CД2 теряют свою способность к оттоку ХС из жировых клеток Ob1771, макрофагов человека и мыши. У них также снижена способность доставлять эфиры ХС в клетки печени через взаимодействие с мембранным рецептором SR-BI [3]. У лиц с преддиабетом обнаружена обратная корреляция между способностью оттока ХС и степенью толерантности к глюкозе [34]. Клиническое исследование EPIC-Norfolk 1745 пациентов с СД2 выявило низкую скорость оттока ХС из макрофагов J774, нагруженных радиоактивно меченным ХС. Отмечена положительная корреляция между оттоком ХС и ХС-ЛПВП/апоА-I и отрицательная корреляция с развитием диабета [35]. Способность ЛПВП осуществлять отток ХС в настоящее время принято считать новым биомаркером, который обратно пропорционален частоте сердечно-сосудистых событий в популяционной когорте. Это открытие показало важность функции ЛПВП по сравнению с оценкой только концентрации ХС-ЛПВП в плазме [36].

Значимое влияние гликирования ЛПВП было отмечено на ABCA1-зависимый транспорт ХС, наблюдалась сильная обратная корреляция между оттоком ХС и гликированием апоА-I по лизину 133, который является частью липидсвязывающего домена апобелка. На модели мышиной диабетической нефропатии, индуцированной стрептозотоцином, было установлено снижение экспрессии транспортеров АBCA1, ABCG1 и SR-B1 [37]. Уровень белка ABCA1 и опосредованный апоА-I отток ХС снижались на 50 и 60% соответственно в перитонеальных макрофагах J774, подвергшихся воздействию гликированного сывороточного альбумина человека, изолированного от пациентов с плохо контролируемым СД 1 и 2 типов [38]. ABCA1-зависимый отток ХС имел обратную корреляцию с HbA1c. Вследствие гликирования изменялась способность апоА-I активировать ЛХАТ (ключевой фермент в обратном транспорте ХС), которая прогрессивно уменьшалась в зависимости от степени гликирования апобелка [28]. Инкубация с метилглиоксалем изменяла экспозицию эпитопа, охватывающего аминокислотные остатки 143–165 апоА-I, участвующие в активации ЛХАТ. При этом специфические моноклональные антитела к этому эпитопу не распознали его. Кроме того, изменялась конформация С-концевого домена апоА-I, где расположены дополнительные эпитопы, регулирующие стабильность апоА-I, связывание с липидами и отток ХС [25]. Гликированный аpoA-I, выделенный из сыворотки крови пациентов с СД2, снижал эффективность связывания и максимальную мощность оттока ХС, при этом Km увеличивалась более чем в 2 раза, Vmax снижалась до 73% [27].

Замена aпoA-I на провоспалительный белок острой фазы SAA также приводила к снижению оттока ХС. При воспалении количество сайтов связывания для ЛПВП, обогащенных SAA, увеличивалось на макрофагах и уменьшалось на гепатоцитах [16]. Кроме того, воспаление вызывало секрецию миелопероксидазы, которая, окисляя апоА-I, приводит к нарушению механизма удаления ХС из макрофагов [39].

Антиоксидантная активность

У пациентов с СД2 окислительный стресс, гликемия, гипертриглицеридемия изменяют структуру ЛПВП, что приводит к снижению их антиоксидантного действия [3][33]. Замена эфиров ХС на ТАГ в липидном ядре ЛПВП значительно изменяет конформацию центрального и С-концевого доменов апоА-I, которые важны для ЛПВП в качестве акцепторов гидроперекисей липидов из ЛПНП [33]. У лиц с метаболическим синдромом и СД2 с нормальными уровнями ТАГ, общего ХС в плазме крови и нормогликемическим фенотипом, но с низкими уровнями ХС-ЛПВП и апоА-I частицы ЛПВП проявляли более низкую антиоксидантную активность (до 43%), чем в группе контроля [40].

Антиоксидантные свойства ЛПВП связаны с присутствием в их составе таких белков, как ПОН-1 и апоА-I. У пациентов с СД1 и СД2 содержание и активность ПОН-1 были вдвое снижены по сравнению с лицами контрольной группы [28][41]. Чем выше интенсивность гликирования ЛПВП-ассоциированных ферментов и апоА-I, тем ниже была активность ПОН-1 и ПОН-3 у пациентов с СД2 при развитии микрососудистых диабетических осложнений [26][41][42].

Предполагают, что потеря активности фермента приходится на более поздние сроки развития СД, но не в стадии резистентности к инсулину [43]. При СД 1 типа концентрации ПОН1 в сыворотке снижается до такой степени, что защита ЛПНП отокисления в условиях in vitro полностью утрачивается, что является признаком плохо контролируемого СД [26]. Низкая активность ПОН-1 может влиять на способность ЛПВП ингибировать окисление как в ЛПНП, так и в самих частицах ЛПВП [2][18]. Cоотношение окисленных ЛПНП/ЛПНП или окисленных ЛПВП/ЛПВП у пациентов с СД2 является биомаркёром окислительного стресса и фактором прогрессирования сосудистых осложнений диабета [2].

В работе Morgantini C. et al. [44] антиоксидантные свойства ЛПВП оценивали как отношение интенсивности флуоресценции ЛПВП + ЛПНП/ЛПНП. Значения индекса ≥1,0 указывало на дисфункциональные ЛПВП (прооксидантные ЛПВП). Значения индекса было значительно выше у пациентов с СД2 по сравнению со здоровыми добровольцами. В результате окисления самих ЛПВП были обнаружены повышенные значения окисленных продуктов арахидоновой и линолевой кислот. Наблюдали положительную корреляцию между антиоксидантным индексом ЛПВП, содержанием окисленных жирных кислот во фракциях ЛПВП и образованием атеросклеротических поражений у пациентов СД2.

Кроме ПОН-1, у лиц с метаболическим синдромом или СД2 значительно снижена активность других, ассоциированных с ЛПВП ферментов, таких как PAF-AH (Platelet-Activating Factor Acetyl Hydrolase) или ЛХАТ из-за их инактивации в результате окисления, гликирования [21][40][45]. Развитие системного воспаления при СД2 сопровождается ростом провоспалительных цитокинов, при этом печень начинает продуцировать маркеры хронического воспаления, С-реактивный белок (СРБ) и SAA, последний замещает апоА-I и ПОН-1 в ЛПВП, что ухудшает их антиоксидантные свойства (рис. 1). Высокие уровни SAA наблюдали у пациентов СД2, замена апоА-I на SAA ухудшала способность ЛПВП защищать ЛПНП от окислительной модификации иих негативного воздействия [44].

Установлено, что ЛПВП, выделенные от здоровых субъектов, значительно снижают продукцию эндотелиального супероксида и активность NADPH оксидазы, стимулированные TNF-α, проявляя мощное антиоксидантное действие ЛПВП на клеткиHAECs. ЛПВП от больных СД2 не оказывали ингибирующего действия на продукцию супероксида или активность NADPH-оксидазы, что указывает на потерю ими антиоксидантных свойств [46].

Про- и противовоспалительные свойства ЛПВП

Противовоспалительные свойства ЛПВП значительно ухудшаются у больных СД2, снижение наблюдали даже у пациентов с хорошим метаболическим контролем, что способствует увеличению риска развития ССЗ, связанных с диабетом [16][47]. Введение реконструированных ЛПВП (рЛПВП, апо А-1 в комплексе с фосфолипидами) пациентам с CД2 увеличивало противовоспалительную способность частиц [22].

В условиях хронического воспаления при СД2 ЛПВП претерпевает множественные структурные изменения, превращаясь в «ЛПВП острой фазы», обогащенные свободными жирными кислотами, ТАГ, SAA и сниженным содержанием противовоспалительных ферментов, включая ПОН-1 [2]. Между значением воспалительного индекса ЛПВП и концентрацией SAA обнаружена статистически значимая корреляция. При замене эфиров ХС на ТАГ и апоA-I на SAA частицы ЛПВП снижали свою активность, как акцепторы окисленных фосфолипидов, что приводило к их накоплению в ЛПНП [44].

Воспалительный индекс ЛПВП можно подсчитать по их способности вмешиваться в миграцию моноцитов, индуцированную ЛПНП. Добавление ЛПВП, полученных от здоровых добровольцев, снижало хемотаксическую активность моноцитов в культуре эндотелиальных клеток HAEC и приводило к уменьшению значения воспалительного индекса ниже 1,0. ЛПВП от больных с СД2 не способны ингибировать миграцию моноцитов, что приводило к росту индекса выше 1,0 [44]. В эндотелиальных клетках ЛПВП ингибируют, вызванное oкисленными ЛПНП, образование MCP1, фактор хемотаксиса моноцитов к очагам воспаления [2].

Липополисахариды, являясь эндотоксинами, индуцируют цитокин-опосредованное системное воспаление. Инактивация эндотоксина ЛПВП осуществляется прямым взаимодействием с апоA-I и включает снижение экспрессии CD14 на моноцитах вкачестве ключевого шага. ЛПВП, связывая липополисахариды, снижают их провоспалительные свойства, что проявляется в ослаблении лихорадки, снижении количества лейкоцитов. У больных с СД2 вклад ЛПВП в нейтрализацию ЛПС снижен и большая часть ЛПС находится в свободном состоянии [3].

ЛПВП могут оказывать противовоспалительное действие путём подавления активации фактора NF-κB в эндотелиальных клетках человека. После предварительной инкубации ЛПВП с культурой HMEC и последующей стимуляцией TNF-α, нативные ЛПВП подавляли фосфорилирование белка p65, необходимого для транслокации фактора NF-κB в ядро и клеточного воспалительного ответа. Напротив, ЛПВП пациентов с СД2 не подавляли активацию NF-κB в этих клетках [48].

ЛПВП, потеряв свой противовоспалительный эффект, начинают проявлять провоспалительные свойства. Так, в клетках HUVEC ЛПВП от больных СД2 усиливали экспрессию мРНК молекул адгезии сосудистых клеток-1 (VCAM-1) в 2-4 раза, при этом уровни CРБ и фактора некроза опухоли-α (TNF-α) повышались. Многопараметрический линейный регрессионный анализ показал, что нарушение противовоспалительной функции ЛПВП было связано с уровнем глюкозы в плазме натощак, концентрацией HbA1c, более низкой активностью ПОН-1 и высокими значениями маркёров хронического воспаления (TNF-α, CРБ) независимо от уровня ХС-ЛПВП [47]. Недостаточная противовоспалительная активность благоприятствует развитию диабета и его осложнений [22].

Влияние на эндотелий сосудов

Многочисленные экспериментальные исследования показали, что ЛПВП оказывают прямое защитное действие на эндотелий сосудов: снижают проявления окислительного стресса, улучшают эндотелий-зависимую вазодилатацию и эндотелиальную репарацию [49]. В эндотелиальных клетках аорты человека HAEC ЛПВП от здоровых лиц активируют эндотелиальную синтазу оксида азота (eNOS) и стимулируют продукцию оксида азота [22]. В связи с гликированием белков ЛПВП и образованием КПГ способность ЛПВП противодействовать вазоконстрикции, вызванной окисленными ЛПНП, активировать продукцию оксида азота была ослаблена у людей с СД2 [48][50]. При этом способность ЛПВП стимулировать eNOS отрицательно коррелировала с концентрацией SAA в ЛПВП и уровнем циркулирующего P-селектина в сыворотке крови, установленного маркера эндотелиальной дисфункции, вырабатываемого эндотелиоцитами под влиянием медиаторов воспаления и провоспалительных цитокинов [48]. У пациентов с СД2 дисфункция эндотелия, зафиксированная по реакциям вазодилатации плечевой артерии, восстанавливалась после введения рЛПВП [51]. Терапия ниацином в течение трёх месяцев у пациентов с СД2 не только повышала уровень ЛПВП, но и, что более важно, противодействовала ингибирующему влиянию окисленных ЛПНП на сосудистую релаксацию [46]. Снижение активации eNOS ЛПВП наблюдалось у пациентов с ожирением и метаболическим синдромом ещё до появления признаков СД [50].

Истощение S1P в ЛПВП, вероятно, является одним из основных факторов, ответственных за снижение сосудорасширяющей функции ЛПВП. Предполагают, что S1P, биоактивный фосфолипид, 50–70% которого переносится в плазме в составе ЛПВП, может напрямую взаимодействовать с эндотелиальными рецепторами S1P, активируя серин/треониновую протеинкиназу В (Akt) и eNOS [50]. Снижение концентрации S1P в ЛПВП у пациентов с СД2 приводило к потере их способности стимулировать фосфорилирование eNOS, уменьшая ее активацию на 40% [48]. У пациентов с метаболическим синдромом ЛПВП были на 39% богаче ТАГ и на 15% беднее S1P, при этом активность eNOS была на 69% ниже. Обогащение S1P ЛПВП пациентам сметаболическим синдромом восстанавливало их способность стимулировать фосфорилирование Akt и активность eNOS [50].

ЛПВП ослабляют апоптоз эндотелиальных клеток, вызванный различными стимулами, такими как TNF-α и oкисленные ЛПНП. Выявлены значительные корреляции между антиапоптотической активностью и содержанием кластерина (apoJ) в ЛПВП. Выживание клеток HAECs (в условиях голодания) дозозависимо увеличивалось при обогащении ЛПВП кластерином за счет ингибирования апоптоза и снижалось при инкубации с антителами против кластерина. Антиапоптотическая активность кластерина, по-видимому, связана с фосфорилированием Akt. Сывороточный уровень кластерина значительно повышается у пациентов с СД2, при развитии коронарной болезни сердца и инфаркте миокарда, что является показателем повреждения сосудов [49].

Метаболизм глюкозы

Известно, что апоA-I/ЛПВП участвуют в регуляции обмена глюкозы, что включает как прямое стимулирующее действие на ее проникновение в скелетные и сердечные мышцы [10][11], так и усиление секреции инсулина [9][10][13].

Гликированный апоА-I утрачивает способность к утилизации глюкозы в глюкозо-толерантном тесте у мышей с инсулинорезистентностью, вызванной диетой с высоким содержанием жиров. Кроме того, aпоA-I, модифицированный метилглиоксалем или гликолевым альдегидом, в составе рЛПВП препятствовал поглощению глюкозы культивируемыми миотрубками скелетных мышц крысы и мыши. Β-клетки поджелудочной железы, предварительно инкубированные с модифицированным апоА-I, показали низкий уровень секреции, стимулированного глюкозой, инсулина по сравнению с нормальным апоА-1 [27]. У пациентов с СД2, получавших внутривенно рЛПВП, происходило снижение уровня глюкозы натощак, улучшилась β-клеточная функция островков по оценке HOMA-β и увеличивалась концентрация инсулина в плазме крови по сравнению с исходным уровнем [11].

Противоопухолевая активность

Эпидемиологические исследования показали, что люди с СД имеют более высокий риск развития колоректального рака, рака молочной железы (РМЖ) и рака мочевого пузыря [52]. Модификации ЛПВП при метаболических заболеваниях, в том числе метаболическом синдроме, ожирении и/или СД2 не только ухудшают их функции, но и способствуют приобретению таких неблагоприятных качеств, как усиление пролиферации, миграции и инвазии клеток рака. Клеточные линии аденокарциномы молочной железы, обработанные дЛПВП, при введении мышам резко повышали процент метастазирования опухолевых клеток в легкие и печень. Гликированные и окисленные ЛПВП стимулировали адгезию клеток аденокарциномы к культуре эндотелиальных клеток HUVEC in vitro, повышали синтез и секрецию сосудистого эндотелиального фактора роста. Для опухолевых клеток адгезия к HUVEC, индуцированная ЛПВП больных РМЖ, осложненных СД2, увеличилась на 33% и 39% по сравнению с ЛПВП от пациентов с РМЖ без диабета и ЛПВП от здоровых лиц, соответственно. При этом происходило увеличение экспрессия ICAM-1 и VCAM-1 и E-селектина эндотелием сосудов, что способствовало начальному метастатическому прогрессированию рака[53].

В экспериментах in vivo также было показано, что у мышей с диабетом db/db количество опухолевых клеток, прилипших к эндотелию сосудов, значительно больше, чем у нормальных мышей C57/BL6. Кроме того, опухолевые клетки во время процесса адгезии располагались в кластерах эндотелия сосудов у мышей db/db в отличие от рассеянного расположения у мышей C57/BL6. По мнению авторов, адгезия связана с повреждением сосудистых эндотелиальных клеток у мышей db/db [53].

## ЗАКЛЮЧЕНИЕ

Результаты многочисленных исследований свидетельствуют о том, что развитие СД2 сопровождается не только снижением уровня ЛПВП в плазме крови, но и существенными изменениями в их структуре. Эти изменения приводят к трансформации нативных ЛПВП в так называемые дисфункциональные или диабетические ЛПВП, которые утрачивают свою способность выполнять антиатерогенные, кардиопротективные и противовоспалительные функции. Такие модификации ЛПВП при СД2 как гликирование, окисление, истощение эфиров ХС и накопление ТАГ, снижение активности ферментов ПОН-1, PAF-AH, ЛХАТ, замена апоА-1 на SAA не только ухудшают их функции, но и способствуют приобретению провоспалительных, проатерогенных свойств, усиливают метастазирование опухоли.

Возможно, что некоторые препараты (ингибиторы БПЭХ или ингибиторы SR-B1) не только увеличивают уровень ЛПВП и ХС-ЛПВП, но могут вызывать появление дисфункциональных ЛПВП у пациентов с диабетом. Например, гиполипидемический препарат торцетрапиб увеличивает содержание апоС-III в ЛПВП, что может быть причиной повышенной продукции медиаторов воспаления и адгезии моноцитов к эндотелиальным клеткам [54]. Напротив, приём статинов (питавастатин) не только повышает уровень ХС-ЛПВП, но также усиливает их антиоксидантные свойства и способность к обратному транспорту ХС [55]. Следует отметить, что у статинов и ниацина может проявляться продиабетогенное действие [56]. Поэтому лечение статинами повышает вероятность развития диабета, если оно назначается людям с факторами риска развития заболевания [57].

В качестве одного из ключевых критериев функциональности ЛПВП предлагается определять способность ЛПВП осуществлять отток ХС из клеток. В настоящее время методические подходы, направленные на выявление дисфункциональных ЛПВП, доступны только научно-исследовательским лабораториям из-за сложности исполнения и отсутствия универсальной стандартной методологии. Успех в поиске биомаркеров дисфункциональности ЛПВП связывают с изучением протеомики и липидома этих частиц [14][58].

Учитывая, что ЛПВП помимо участия в транспорте ХС, выполняют в организме важные регуляторные функции [59], есть основание предполагать, что структурная модификация ЛПВП при диабете является одной из причин развития сердечно-сосудистой патологии и высокой смертности. Терапевтические подходы, направленные на предотвращение этих осложнений, могут включать использования антиоксидантных препаратов, предотвращающих окислительную модификацию ЛПВП, атакже повышение уровня ЛПВП/апоА-I с помощью генной терапии или введения в организм реконструированных ЛПВП и рекомбинантных апоА-I-миметиков [60].

## ДОПОЛНИТЕЛЬНАЯ ИНФОРМАЦИЯ

Источники финансирования. Публикация подготовлена в рамках выполнения государственного задания, регистрационный номер 122032300152-3

Конфликт интересов. Авторы декларируют отсутствие явных и потенциальных конфликтов интересов, связанных с публикацией настоящей статьи.

Участие авторов. Потеряева О.Н.— поиск и анализ данных литературы, написание статьи; Усынин И.Ф.  — поиск данных литературы, редактирование текста, внесение ценных замечаний. Все авторы одобрили финальную версию статьи перед публикацией, выразили согласие нести ответственность за все аспекты работы, подразумевающую надлежащее изучение и решение вопросов, связанных с точностью или добросовестностью любой части работы.
